# Exploring the spatial distribution of biomolecules in *Pseudomonas aeruginosa* biofilms formed on PDMS and lung explants by mass spectrometry imaging

**DOI:** 10.1016/j.bioflm.2026.100401

**Published:** 2026-09-17

**Authors:** Marie Droniou, Clément Guillou, Hung Le, Christophe Arnoult, Teddy Grandjean, Philippe Gosset, Pascal Cosette, Isabelle Schmitz

**Affiliations:** a Univ Rouen Normandie, INSA Rouen Normandie, CNRS, Normandie Univ, PBS UMR 6270, Rouen, France; b Univ Rouen Normandie, INSERM US 51, CNRS UAR 2026, HeRacLeS PISSARO, Rouen, France; c Univ Rouen Normandie, Université Caen Normandie, Normandie Univ, CBSA UR 4312, Rouen, F-76000, France; d Univ. Lille, CNRS, INSERM, CHU Lille, Institut Pasteur de Lille, U1019-UMR 9017, Center for Infection and Immunity of Lille, Lille, France

**Keywords:** *Pseudomonas aeruginosa*, Bacterial biofilm, Mass spectrometry imaging (MSI), Polydimethylsiloxane (PDMS), Lung explant

## Abstract

Bacterial biofilms constitute a form of collective organization that enhances bacterial tolerance to both antibiotics and immune defences. Among bacteria, *Pseudomonas aeruginosa (P. aeruginosa)* is a major opportunistic pathogen, with marked biofilm-forming capacity, highly implicated in chronic pulmonary infections. Understanding the metabolic and structural dynamics of *P. aeruginosa* biofilms is thus essential for the development of new therapeutic strategies. In this work, we investigated the formation of *P. aeruginosa* biofilm and its molecular composition on two different substrates of importance in public health. The first model is a synthetic polydimethylsiloxane (PDMS) surface, an inert model commonly used to simulate biomedical devices, and the second is a murine lung explant which offers a more physiologically relevant context to study lung infection. Using these models, mass spectrometry imaging (MSI), specifically MALDI-MSI technology, was used to map the spatial distribution of biofilm-produced compounds without prior labelling. This approach enabled the detection of multiple molecular families involved in the regulation, communication and structural organization of biofilm. The technique provides additional information on differences in the distribution of these molecules, particularly phenazine derivatives, within the models. Indeed, biofilms formed on lung explants show increased production of pyocyanin, invading lung tissue. These results highlight the power of MSI to explore the spatial metabolism of bacterial biofilms. It opens interesting perspectives for the microbiology of chronic infections by enabling visualization of chemical interactions between pathogens and their host environment.

## Highlights

• Optimization of two tailored biofilm models for mass spectrometry imaging.
• Mapping of molecular distributions within *Pseudomonas aeruginosa* biofilms.
• Deep pyocyanin penetration into lung tissue.

## 1 Introduction

Bacterial biofilms represent a ubiquitous and highly adaptive form of microbial organization [1]. These structures, composed of bacterial communities embedded in an extracellular matrix of polysaccharides, proteins, and extracellular DNA, allow microorganisms to persist in hostile environments, whether natural, industrial, or clinical [2,3]. Cells within biofilms exhibit increased tolerance to antimicrobial agents and host immune defences, thus contributing to the chronicity of many infections [4,5]. In the medical context, this enhanced survival capacity confers to biofilms a major role in the persistence of infections associated with medical devices and infected tissues.

Among pathogenic biofilm-forming bacteria, *P. aeruginosa* is a leading model organism. This opportunistic pathogen is responsible for severe nosocomial infections and is particularly feared in patients with cystic fibrosis, where it colonizes the respiratory tract and forms biofilms resistant to mucus decongestant and antibiotic treatment [6]. In the lungs, *P. aeruginosa* develops dense biofilm structures that promote bacterial cell protection, intercellular communication via quorum sensing (QS) systems, and modulation of the host immune response [7]. Studying these biofilms in a pulmonary context is therefore essential to understand the molecular mechanisms underlying infection persistence. However, the inherent structural and chemical heterogeneity of biofilms remains a major challenge for their study [8]. The diverse microenvironments found within biofilms generate physiological heterogeneity among bacterial subpopulations which is difficult to capture using conventional analytical approaches.

In this context, next-generation spatial and chemical analysis techniques, such as MSI, offer unique perspectives for the study of biofilms. This technique allows the mapping of the distribution of biomolecules (metabolites, lipids, peptides) directly on samples, without prior labelling [9,10]. MSI, whether based on matrix-assisted laser desorption/ionization (MALDI-MSI), solvent beam ionization (DESI-MSI), or secondary ion mass spectrometry (SIMS), provides both spatial and chemical information on biofilm composition [11]. These approaches can give essential information on localization of the metabolites, in addition to global methods such as the study of Zhang et al. [12], that worked on interior and periphery bacteria extraction methods to give information on metabolic gradients, intercellular interactions, and molecular communication phenomena within bacterial biofilms.

Despite the rise of MSI to study these microbial communities, the analysis of biofilms remains a significant analytical and spatial challenge due to their fragile structure and molecular heterogeneity. Sample preparation is therefore a critical step, requiring methods that preserve the integrity of biofilms while minimizing metabolite delocalization. In particular, the choice of embedding medium is crucial, as it can significantly influence ionization efficiency and introduce competition ionization effects.

Previous studies have successfully adapted MSI for the study of bacterial colonies, highlighting the spatial distribution of metabolites involved in QS, virulence, antibiotic response mechanisms and interspecies or host-microbe interactions [[13], [14], [15]]. However, most MSI studies focus on bacterial colonies formed on agar, which only partially reflect the structural organization and the surface-associated growth conditions of biofilm. MSI-compatible biofilm models that both preserve architecture and maintain analytical performance remain limited, particularly to study host-biofilm interactions.

The experimental models used to study biofilms also play a crucial role in the biological relevance of the results obtained. Synthetic surfaces, such as PDMS, are widely used in biomedical applications owing to their biocompatibility, optical transparency, and ease of structuring [16]. As such, PDMS is an interesting model to simulate biomaterial-bacteria interfaces, such as those found on implantable medical devices or catheters [17]. Conversely, lung explants offer a more physiological *ex vivo* environment, allowing the study of direct interactions between bacteria and host tissues [18]. This model more accurately reflects tissue conditions, including extracellular composition and the presence of immune mediators. Therefore, comparing biofilms grown on different substrates allows to assess the influence of surface properties on the structural and metabolic organization within biofilms. Although biofilm models are essential to study bacterial metabolism and host interactions, most of the techniques don't allow for a precise spatial analysis.

Here, our study aims to characterize and compare *P. aeruginosa* biofilms grown on PDMS surfaces and lung explants using MSI. This comparison was performed to reveal the molecular signatures associated with the bacterial adaptation to diverse environments and contribute to a better understanding of tissue colonization mechanisms. Specifically, we first developed and optimized biofilm culture, sectioning and embedding on these two substrate types. Then, we applied MSI techniques to map the spatial distribution of key metabolites. The results will lead to a better understanding of biofilm-surface and biofilm-tissue interactions.

## 2 Material and methods

### 2.1 Bacterial strains and biofilm formation

#### 2.1.1 Bacterial strains and growth conditions

For microbiological experiments, the strains of *P. aeruginosa* PAO1-eGFP and PA14 were used. They were stored in Mueller Hinton (MH) (Difco™) medium supplemented with 20% glycerol at −80°C. Before each experiment, 5 mL of MH was inoculated from a single colony of *P. aeruginosa* strain, previously cultivated on MH agar plates, and grown overnight (16 h, 37°C, 147 rpm shaking).

Before initiating biofilm formation on the different substrates, overnight bacterial cultures were diluted to obtain an initial density of 10^6^ CFU/mL in different culture media for comparison: standard MH medium and artificial sputum medium (ASM) mimicking the cystic fibrosis lung environment. ASM was prepared by mixing 1.75 g of NaCl, 0.77 g of KCl, 0.64 g of Tris Base, 1.75 g of casein, 1.4 g of fish sperm DNA and 1.75 g of mucin in 350 mL of deionized water.

#### 2.1.2 Preparation of substrates and biofilm formation

##### 2.1.2.1 Preparation of PDMS surfaces

PDMS surfaces were produced using Sylgard 184 kit (Ellsworth Adhesives) as described previously [17]. Briefly, 12 g of elastomer base was mixed with 1.2 g of curing agent (10:1 ratio) and poured into a flat Petri dish. Air bubbles were removed under vacuum before reticulation at 55°C overnight. The cured PDMS was then cut into 1 cm^2^ square surfaces with a scalpel and sterilized under UV light for 15 min. PDMS surfaces were stored at room temperature. The PDMS surfaces were transferred into individual wells of a 24-well plate.

##### 2.1.2.2 Lung explants preparation

The lungs were obtained from fresh cadavers of healthy mice aged 10 to 12 weeks. After gas anesthesia with 5% isoflurane in air, the mice were euthanized by cervical dislocation, the lungs were removed and immersed in phosphate-buffered saline (PBS) at 4°C. Lung lobes were immediately dissected and cut into squares (approximately 0.5 × 0.5 cm; ∼25 mg/piece). The tissue pieces were washed once with PBS and transferred into individual wells of a 24-well plate containing 200 μL of 1.7% (w/v) solidified agarose at the bottom.

#### 2.1.3 Biofilm formation

PDMS surfaces were inoculated with 1 mL of 10^6^ CFU/mL of *P. aeruginosa* PAO1-eGFP or PA14 culture prepared in MH or ASM medium and incubated for 24 h at 37°C under static conditions. For lung explants, tissues were inoculated with 1 mL of 10^6^ CFU/mL of PAO1-eGFP culture prepared in ASM and incubated for 24 h at 37°C, 5% CO_2_, under static conditions.

### 2.2 Biofilm embedding and slicing

#### 2.2.1 Mass spectrometry compatibility assay

To assess the compatibility of embedding media with MSI procedure, particularly their ability to induce unwanted signals, four embedding media were tested in MSI experiments. 1 μL of agarose (3%), carboxymethyl cellulose (CMC) (5%), Optimal Cutting Temperature compound (OCT) or gelatin (10%) was dropped onto adhesive slides and 2,5-dihydroxybenzoic acid (DHB) matrix was deposited with SMALDI Prep (TransMIT GmbH) equipment (30 mg/mL in acetone/water 1:1, 0.1% trifluoroacetic acid (TFA)). 25 scans were acquired in positive ion mode at two *m/z* mass ranges, 100-500 and 500-1500.

#### 2.2.2 Embedding assay

To further evaluate embedding media, CMC and gelatin were tested on a lung explant sample to evaluate their capacity to preserve sample integrity without affecting analyte ionization. Briefly, the CMC solution was prepared by dissolving 5 g of CMC powder into 100 mL of water. Besides, 20 g of gelatin was dissolved in 100 mL of water and heated to 100°C to obtain dense gelatin which was subsequently cooled to room temperature before embedding. Lung pieces were put in 24-well plate containing 200 μL of pre-warmed semi-solidified agarose (1.7%). Once the agar had solidified, 1 mL of gelatin (10%) or CMC (5%) was added to embed the lung fragments and the plate was then placed at −20°C overnight. Embedded lungs samples were collected and mounted onto the sample holder with Tissue Freezing Medium (Leica) and sliced at 20 μm thickness with a cryostat (Leica CM3050S) at −21°C. The lung sections were collected onto adhesive microscopy slides (Epredia, Superfrost Plus), dried in a desiccator at room temperature for 30 min and stored at −20°C until analysis.

##### 2.2.2.1 Analytes ionization

For each condition of embedding, 2 slices were sprayed with DHB matrix (30 mg/mL in acetone/water 1:1, 0.1% TFA) using SMALDIPrep equipment (10 μL/min for 14 min). The DHB matrix solution was prepared with 17:0 lysophosphatidylethanolamine (40 μg/mL) (Avanti), leucine enkephalin (2.8 μg/mL) (Sigma-Aldrich), ampicillin (110 μg/mL) (Sigma-Aldrich), 1-pentadecanoyl-2-hexadecanoyl-glycero-3-phospho-(1′-sn-glycerol) PG 15:0 (40 μg/mL) (Avanti) and 2-fluorophenylglycin (50 μg/mL) (Fluorochem) as internal standards. For comparison, 5 acquisitions of 100 scans each were performed in positive ion mode, at the interface between the lung and embedding medium. The signal intensity of each internal standard was then compared in CMC and gelatin embedding conditions to evaluate potential matrix effects on analyte ionization.

##### 2.2.2.2 Tissue integrity evaluation

To evaluate the integrity of lung samples depending on the embedding media, the other half of slices was stained with hematoxylin-eosin (H&E) coloration. Briefly, slides were first fixed in 70% ethanol for 5 min and washed in water for 3 min. Slides were then stained with hematoxylin for 1 min, followed by a wash under running water for 3 min. Subsequently, slides were immersed in eosin for 30 s and washed 70% ethanol (10 dips) before dehydration in 100% ethanol for 4 min (2 min in two different baths). Slides were placed in xylene mixture (Sigma-Aldrich) for 10 min and mounted onto a glass microscope slide using Entellan (Sigma-Aldrich) mounting medium. Slides were observed with widefield color microscopy (Leica Thunder Tissue 3D) after adding the mounting medium for at least 4 h.

#### 2.2.3 Biofilm embedding and slicing

After 24 h of biofilm formation, half of culture medium was gently removed and replaced with a gelatin solution (20%). Plate was left at room temperature for 10 min and then placed in a −20°C freezer overnight to allow complete solidification.

#### 2.2.4 Biofilm slicing

Embedded surfaces were collected and PDMS surfaces were removed from frozen gelatin block. The remaining gelatin blocks containing biofilm, and lung explants were mounted onto the sample holder with a minimal quantity of Tissue Freezing Medium (Leica) and were sliced at 100 μm and 20 μm thickness with a CM3050S cryostat (Leica Biosystems, Wetzlar, Germany) at −21°C. Biofilm sections were collected onto adhesive microscopy slides (Epredia, Superfrost Plus) or classic microscopy slides and stored at −20°C. To increase biofilm surface coverage during acquisition, the cutting plane was adjusted as presented in Fig. 3B.

Prior to any downstream analysis, biofilm sections were placed in a desiccator for 30 min to prevent condensation upon removal from −20°C storage.

### 2.3 Histology and microscopy

#### 2.3.1 Fluorescent labelling on PDMS surfaces

Bacterial biofilms formed on PDMS surfaces were stained with SYTO 9 (Thermo Fisher Scientific) and slices were mounted onto a glass microscope slide using Mowiol 4-88 mounting medium. Image acquisition was carried out using a Leica Thunder Tissue 3D widefield microscope. SYTO 9 was excited at 488 nm and fluorescence emission was collected with a band-pass filter set at 500-540 nm for SYTO 9. Image analysis was performed using ImageJ software.

#### 2.3.2 Histology and immunofluorescence on lung explants

Lung explants were stained with H&E staining as described above. Consecutive slides were used to conduct immunofluorescence experiments. The latter were performed with primary antibodies directed against *P. aeruginosa* (Sigma-Aldrich) and fluorescent secondary antibodies (Sigma-Aldrich) (excitation at 488 nm). Briefly, 100 μL of primary antibodies was dropped onto lung slices for 1 h at 4°C after formalin fixation for 1 h at room temperature. Slices were washed once with PBS between and after those two steps. Subsequently, 100 μL of secondary antibodies (anti-rabbit) coupled with a 488 nm fluorochrome were added for 1 h at 4°C and washed once with PBS. Slices were stained with ActinRed and DAPI and mounted onto a glass microscope slide using Mowiol 4-88 mounting medium. Slides were observed with a Leica Thunder Tissue 3D widefield microscope. DAPI, fluorescent antibodies and ActinRed were excited at 405, 488 and 552 nm, respectively. Fluorescence emission was collected sequentially with band-pass filters set at 450-480 nm for DAPI, 500-540 nm for coupled antibodies and 570-620 nm for ActinRed. Image analysis was performed using ImageJ software.

### 2.4 Application of MALDI-MSI to visualise biomolecules within biofilms

#### 2.4.1 Matrix application

A matrix solution of 30 mg/mL DHB was prepared in acetone/water 1:1, 0.1% TFA before each matrix application. Then, a total of 140 μL of DHB solution was sprayed onto biofilm sections at a flow rate of 10 μL/min, with a sample holder rotation speed of 500 rpm (SMALDIPrep, TransMIT GmbH, Giessen, Germany).

#### 2.4.2 MALDI-MSI acquisition

All measurements were performed using an autofocusing AP-SMALDI5 high-resolution MALDI imaging ion source (TransMIT GmbH, Giessen, Germany) coupled to a hybrid quadripolar-Orbitrap analyzer (Q Exactive Plus, Thermo Scientific).

Laser parameters were set to 100 Hz and 50 laser pulses per pixel. The spatial resolution was 5 μm for biofilm formed on PDMS and 20 μm for biofilm formed on lung explant, with a laser attenuation of 39°. All MSI experiments were performed in positive-ion mode with a mass resolution of 140.000 in the *m/z* 200, at 100-1200 *m/z* mass range. External mass calibration was realized prior to each set of experiments with *m/z* ions characteristic of the DHB matrix. Internal mass calibration was set by using a lock mass at *m/z* 545.07145 (corresponding to 4 M + H-4H_2_O + DHB cluster). Mass measurement accuracy was checked on known metabolites and was found to be better than 3 ppm. Ion injection time was set to 500 ms, S-lens level to 90 and capillary temperature was set to 350°C.

#### 2.4.3 Matrix-matched standard spotting validation

The ionization behaviour and the specificity of spatial localization of four standards, the Pseudomonas Quinolone Signal (PQS) (Sigma-Aldrich), 2-heptyl-4-hydroxyquinoline-N-oxide (HQNO) (Sigma-Aldrich), 2-heptyl-4-quinolone (HHQ*)* (Sigma-Aldrich) and pyocyanin (Sigma-Aldrich) were assessed under the experimental conditions used for MALDI-MSI. Briefly, authentic standard of each metabolite was first evaluated on MALDI slides at four concentrations (5, 50, 150 and 300 μM, diluted in methanol/water 4:1). 1 μL was deposited onto the slide, dried for 30 min and coated with DHB matrix using the same procedure described in “Matrix application” section. Based on these experiments, a suitable concentration was selected for matrix-matched spotting experiment.

For matrix-matched validation, 0.2 μL of each corresponding standard at 50 μM was spotted onto a control lung section of *ex vivo* model. The experiment was performed across three consecutive lung sections by swapping the positions of the four standards to avoid tissue variability. All sections were acquired in MALDI-MSI to evaluate the *m/z* and spatial localization of each standard. Moreover, MS/MS was performed *in situ* for each standard to confirm metabolite annotation and absence of intense isobaric ions from the biological matrix.

#### 2.4.4 MSI data treatment and analysis

All spectra were treated using SCILS™ Software (Bruker). Ion density maps were built for each selected ion after root mean square normalization. Co-localization analysis was performed to further explore spatial relationships between molecular species. Quantitative analysis was performed by extracting ion intensities from predefined regions of interest. Briefly, different regions of interest were designed: superficial or deep biofilm layers and interface with PDMS for *in vitro* model (Fig. 5A) and infected and uninfected lung tissue for *ex vivo* model (Fig. 5B). Analysis was performed across three independent biological replicates (n = 3) for both models. For each feature, the signal distribution in each region was expressed as a percentage of the total signal.

Metabolites were annotated based on the determination of molecular formulae thanks to the high mass measurement precision, based on METASPACE database [19] search and based on MS/MS experiments when possible. Annotation on METASPACE was performed using a *m/z* tolerance of 3 ppm with original MSM version from HMDB-v4, SwissLipids-2018-02-02, LipidMaps-2017-12-12 and PAMDB-v1.0 databases. MS/MS experiments were performed *in situ* on *in vitro* and *ex vivo* model slices by selecting the targeted precursors from MS spectra. Precursor ions were fragmented in positive ion mode with a normalized collision energy ranging from 15 to 60%, depending on the precursor ion. Annotation levels were detailed based on the Metabolomics Standards Initiative definition [20,21].

## 3 Results and discussion

**Fig. 1:**
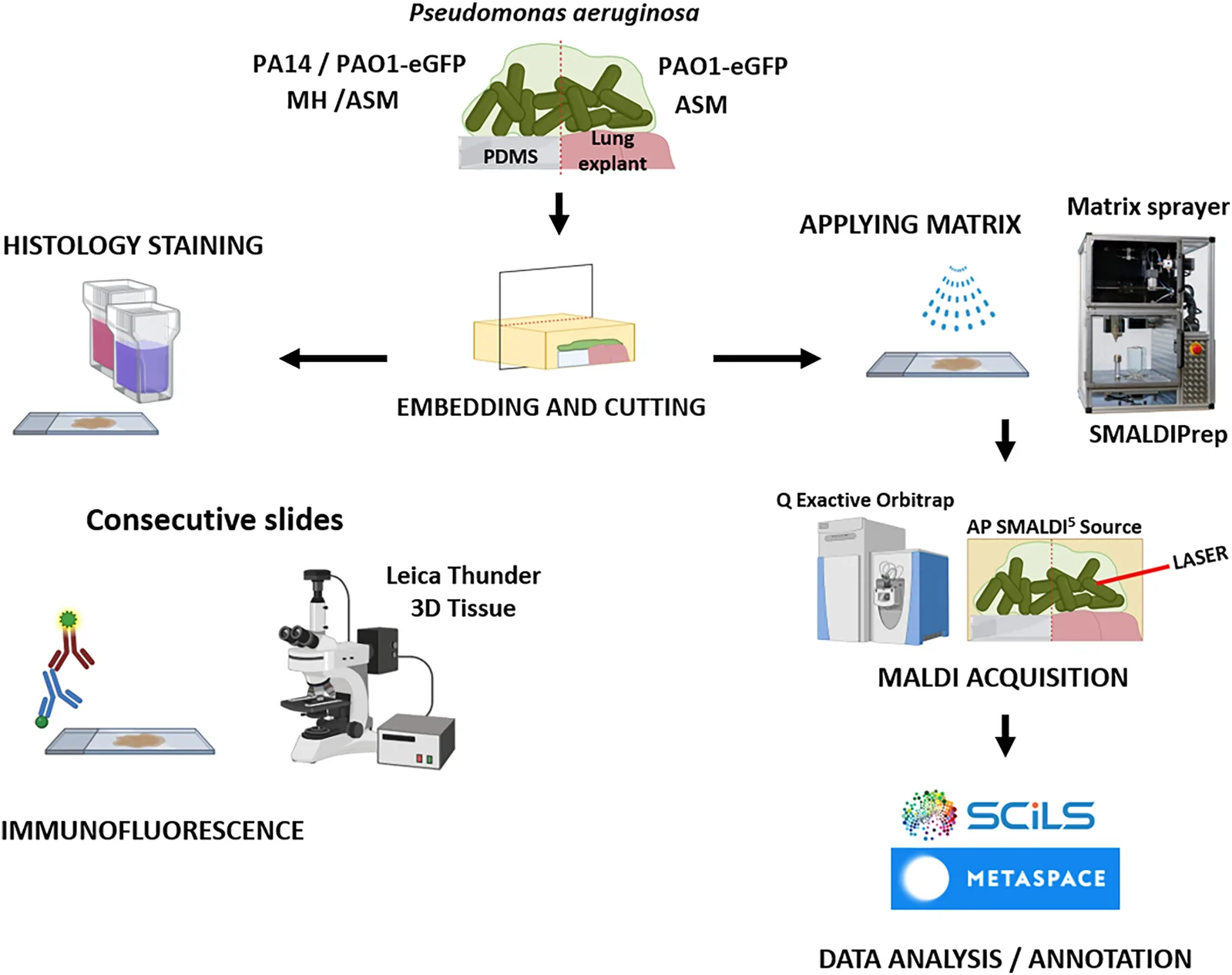
Schematic workflow to explore the spatial distribution of biomolecules in***P. aeruginosa*biofilms formed on PDMS and lung explants using mass spectrometry imaging.***P. aeruginosa* biofilms were grown separately on two different substrates. PDMS surfaces were inoculated with PA14 or PAO1-eGFP strains on MH or ASM media. Lung explants were inoculated with PAO1-eGFP strain in ASM medium.

This study aimed to investigate the metabolic spatial organization of *P. aeruginosa* biofilms using MSI in two complementary experimental models: an *in vitro* biofilm formed on a PDMS surface and an *ex vivo* lung explant model designed to mimic host colonization. Prior to MSI analyses, several methodological parameters were optimized to preserve biofilm architecture and tissue integrity during cryosectioning. These optimizations included the evaluation of different embedding media, based on MSI signal intensity. These also included the choice of biofilm growth conditions and sectioning strategies, based on biofilm thickness to maximize the spatial information accessible during MSI acquisition (Fig. 1).

### 3.1 Gelatin generates the lowest level of interfering ions in MSI analyses

Given the structural fragility of biofilms, selecting an appropriate embedding medium is essential to preserve their integrity during cryo-sectioning and subsequent MSI analysis. Previous MSI studies mostly analyzed intact biofilms and therefore mainly provided information on metabolites present at the surface. Only a few studies have investigated biofilm sections to reveal metabolic heterogeneity across biofilm depth. For example, Wakeman et al. [22] analyzed interactions between *P. aeruginosa* and *Staphylococcus aureus* in the presence of calprotectin using biofilms frozen in 25% OCT or Milli-Q water and sectioned at −20°C. Another study by Rivera et al. [23] investigated lipid distributions across *S. aureus* biofilm layers using a CMC/gelatin embedding mixture.

**Fig. 2:**
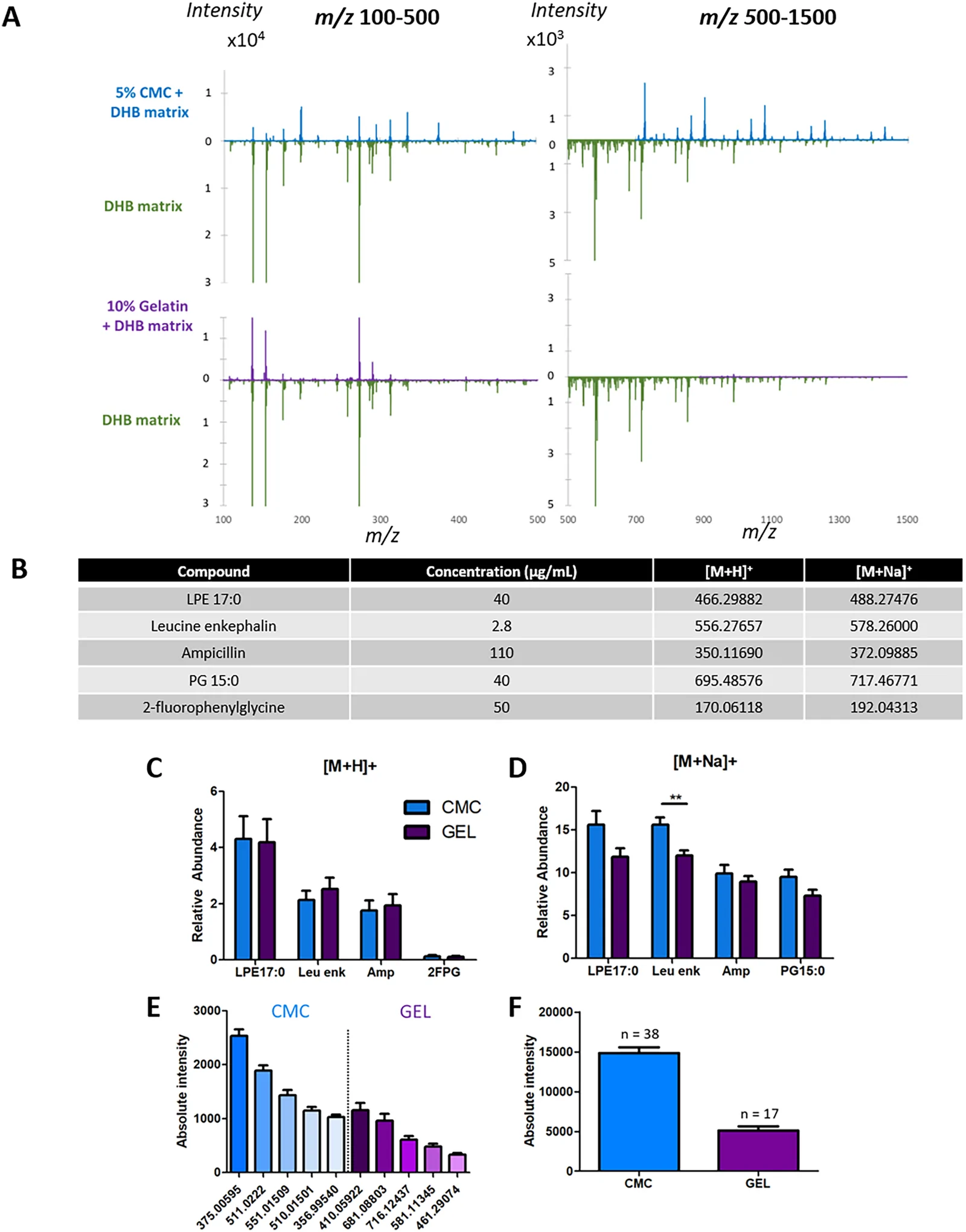
Optimization of biofilm embedding. A: Mirror plot comparing CMC and gelatin mass spectra under MALDI conditions with DHB matrix and DHB spectra alone at *m/z* 100-500 and *m/z* 500-1500 mass range. B: List of internal standards used for comparison. C-D: Internal standards intensity in CMC or Gelatin sample, with [M+H]^+^ or [M+Na]^+^ at lung-embedding medium interface. E: Absolute intensity of 5 more intense CMC or GEL specific ions at lung-embedding medium interface. F: Absolute intensity of 38 CMC and 17 GEL specific ions at lung-emdedding medium interface.

The selection of an appropriate embedding medium is a critical step for MSI analysis of biofilms because interfering ions generated by the embedding matrix can suppress analyte signals. Here, to identify a suitable embedding medium for MSI analysis, four commonly used media were evaluated for potential signal interference in the *m/z* 100-1500 range: OCT [22], agar [24], gelatin [25], and carboxymethyl cellulose (CMC) [26]. As shown in Fig. 2A and Supplementary Fig. S1, OCT generated numerous peaks between *m/z* 1000 and 1500 corresponding to polyethylene glycol (PEG) polymers. Agar also produced several intense interfering peaks and showed poor embedding properties. In contrast, gelatin and CMC generated fewer interfering ions and showed limited signal suppression, which may improve detection of analytes within the studied mass range. This result is consistent with previous studies conducted on MALDI analysis in MSI, where the two most commonly used coating media are CMC and gelatin which have already been used simultaneously for biofilm embedding [23].

To validate these observations under experimental conditions relevant to the model, interference and signal suppression were also assessed directly on lung sections. Five standard molecules (Fig. 2B) were sprayed with the matrix and analyzed at the interface between lung and embedding medium using only gelatin or CMC (five replicates per slide). The relative abundances of the standards were comparable between both embedding media, and no significant difference in ion intensity was observed (Fig. 2C and D). However, the number and the relative intensity of interfering ions differed between the two media. The most intense ions detected in gelatin were approximately three times less intense than those detected in CMC (Fig. 2E and F). Based on these results, gelatin was selected as the embedding medium for this study. This result is supported by a previous study seeking the most suitable coating medium to coat living organisms [27].

Histological evaluation of lung sections was also performed in parallel and confirmed the suitability of gelatin (Fig. 4A). Lung sections embedded in gelatin and stained with H&E showed a well-preserved interface between the lung tissue and the embedding medium, demonstrating that gelatin maintained tissue integrity during cryo-sectioning.

### 3.2 Optimization of culture conditions and sectioning strategy enhances biofilm surface area

**Fig. 3:**
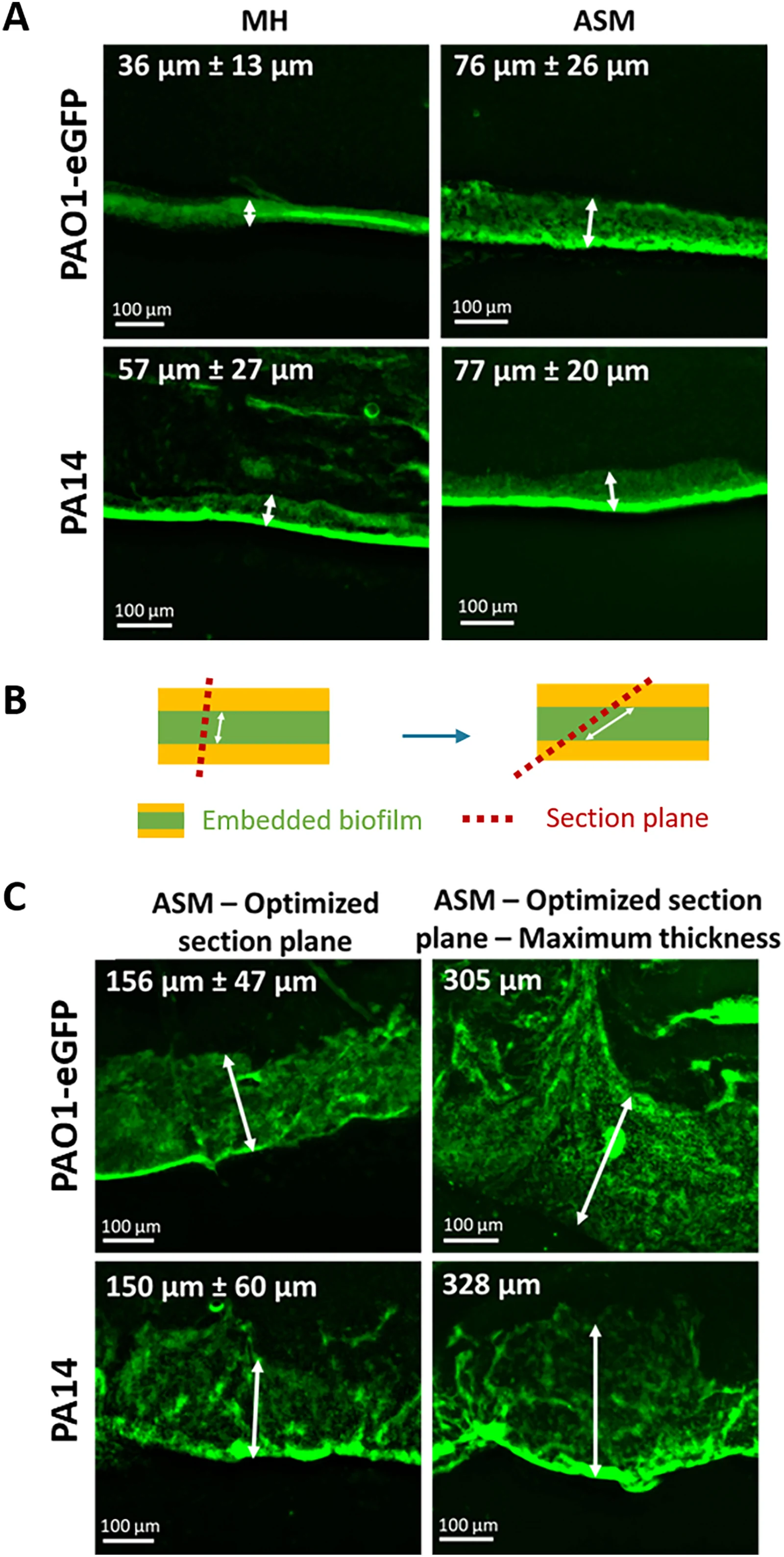
Comparison of biofilm thickness depending on***P. aeruginosa*strain and culture medium and section plane.** All slides were stained with SYTO 9 and mounted onto a glass microscope slide using Mowiol 4-88 mounting medium. A: Average thickness and standard deviation of PAO1-eGFP and PA14 biofilm cultivated in Mueller Hinton or artificial sputum medium. B: Optimization of section plane to increase biofilm thickness and to improve biofilm visualization in MSI. C: Average and standard deviation and maximum thickness of PAO1-eGFP and PA14 biofilm cultivated in Mueller Hinton or Artificial Sputum medium cut with optimized section plane. Biofilm thickness is highlighted by white arrows. Scale: 100 μm.

To optimize MSI analysis of biofilms, *P. aeruginosa* biofilm models were developed under different conditions. Biofilms were first cultivated on PDMS surfaces to mimic colonization of implanted medical devices. PDMS surfaces were inoculated with either PAO1-eGFP or PA14 strains and cultivated in two different media: MH and ASM. Following growth, biofilms were embedded in 10% gelatin, frozen at −20°C, cryo-sectioned, and stained with SYTO 9 to evaluate biofilm thickness by fluorescence microscopy (Fig. 3).

Biofilm thickness measurements showed that biofilms grown in ASM were thicker than those grown in MH medium. The average thickness reached approximately 77 μm, corresponding to an increase of about 30%. This observation is consistent with previous studies showing that ASM promotes biofilm formation due to its composition, which includes amino acids and components mimicking extracellular DNA [28]. Extracellular DNA is known to contribute to biofilm structural stability through interactions with matrix components [29]. In addition, the higher viscosity of ASM may further facilitate the formation of thicker and more structurally organized biofilms.

Both PAO1-eGFP and PA14 strains formed biofilms of similar average thickness in ASM, although PA14 biofilms appeared more homogeneous (Fig. 3A). While both strains produced comparable biofilm thickness after 24 h, they are known to rely on different mechanisms during biofilm initiation and development. PAO1 typically accumulates biomass rapidly on uncolonized surfaces, whereas PA14 is more efficient at invading existing biofilms [30]. Because the present experiments focused on mature biofilms, these early-stage differences were not expected to strongly affect the final thickness measurements.

Given the 5 μm spatial resolution of the MALDI laser, an 80 μm-thick biofilm would be represented by only 16 pixels across its depth, limiting the spatial information that can be extracted. Maximizing the MSI acquisition area would therefore improve the assessment of biofilm heterogeneity. Instead of increasing biofilm thickness, the cutting orientation was modified to increase the exposed biofilm surface (Fig. 3B). Microscopy confirmed that the optimized sectioning plane doubled the acquisition area, reaching lengths greater than 300 μm. This modification significantly increased the number of pixels that could be analyzed across the biofilm (Fig. 3C).

### 3.3 *P. aeruginosa* invades and degrades the respiratory epithelium *ex vivo*

**Fig. 4:**
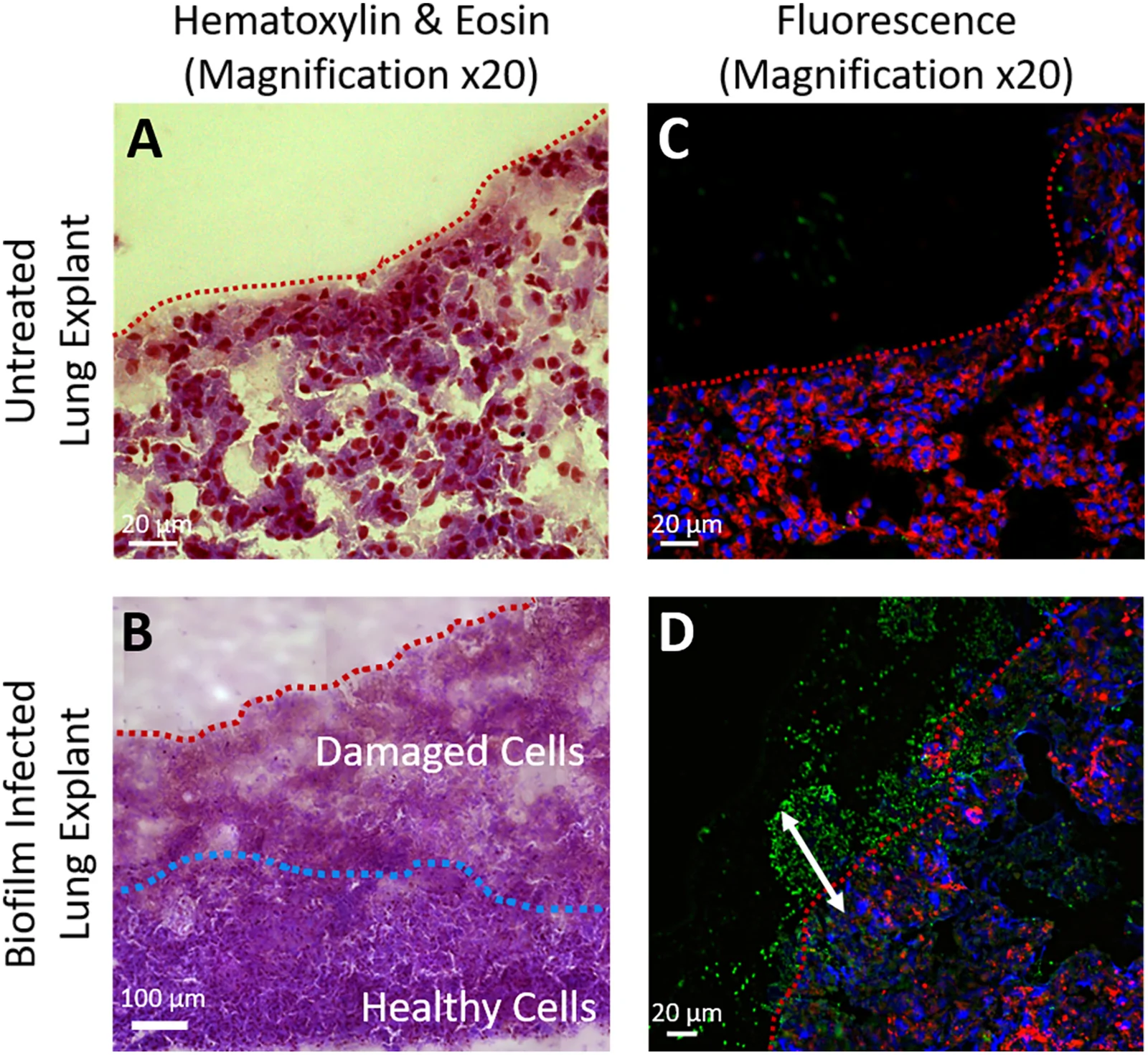
Visualization of biofilm formed on lungexplants. A: H&E staining of control lung explant indicates the maintenance of tissue integrity after embedding with gelatin. B: Infected lung explant stained with H&E staining reveals an area with damaged cells and an area not yet affected by the infection delimited by the dotted blue line. C-D: Immunofluorescence and DAPI/Red-Actin staining was conducted onto consecutive control or infected lung explant slides. The red dotted lines represent the surface of the lung explant. Biofilm is represented with white arrow. Actin: red, DAPI: blue, *P. aeruginosa*: green.

An *ex vivo* model was also developed to investigate host/biofilm interactions (Fig. 4). Lung explants were inoculated for 24 h with the PAO1-eGFP strain in ASM medium and processed using the same embedding and cryosectioning procedure as *in vitro* model. Histological analysis using H&E staining revealed good preservation of the lung structure after embedding (Fig. 4A). After 24 h of infection, cells located at the lung surface showed clear signs of damage in the presence of the biofilm, including progressive disruption of cellular architecture and loss of nuclear structures (Fig. 4B). This progressive damage results in a distinct interface between damaged and healthy cells within the tissue (Fig. 4B).

Immunofluorescence staining using antibodies directed against *P. aeruginosa*, combined with actin and DAPI markers for lung cells, was performed in the absence (Fig. 4C) or the presence of bacteria (Fig. 4D). These analyses confirmed the presence of PAO1-eGFP biofilms on the lung surface. As histological analysis, fluorescence imaging of infected samples revealed the disruption of tissue organization, characterized by a marked loss of actin staining or diffuse actin signal. Moreover, although DAPI blue fluorescence was detected, no distinct nuclear structures could be identified, resulting in a non-specific blue signal. Bacterial cells were also detected in deeper layers of the lung explant, demonstrating the invading capacity of *P. aeruginosa*. These observations are consistent with previous reports describing the ability of *P. aeruginosa* to degrade and invade respiratory epithelium during infection [31].

### 3.4 MALDI-MSI reveals metabolite distribution within biofilms

**Fig. 5:**
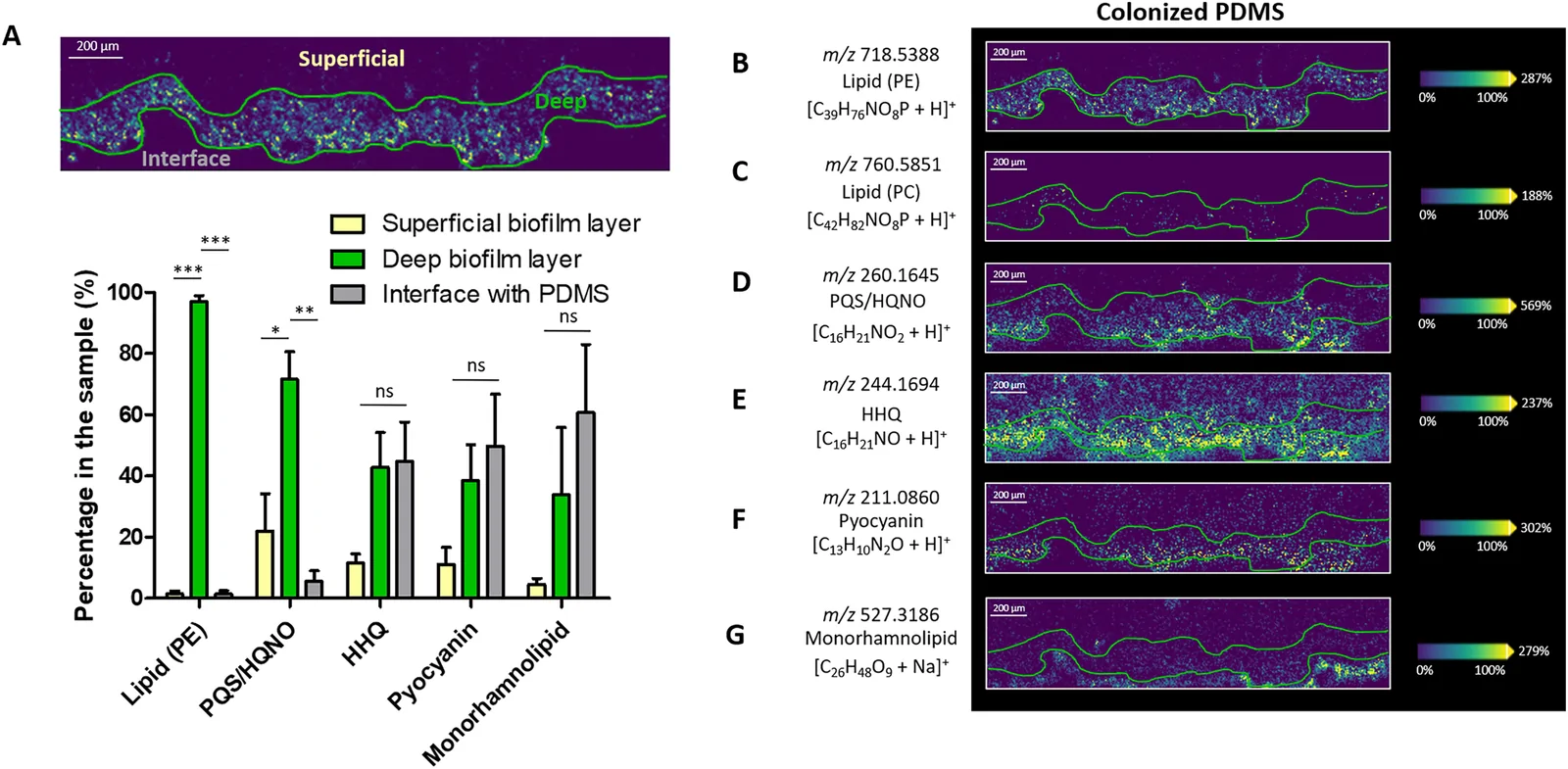
Spatial distribution of six metabolites involved in***P. aeruginosa*biofilm on PDMS surface.** (A) Schematic representation of the three biofilm layers on PDMS surface and percentage of each ion in the three biofilm layers on PDMS surface. (B-G) Representative density ionic maps of six ions [M+H]^+^ (B-F) and [M+Na]^+^ (G) detected in *P. aeruginosa* biofilm formed on PDMS after 24h. Data are presented as mean ± SEM; Statistical analysis (n = 3) were performed using one-way ANOVA with a Bonferroni post-test; ns: not significant; *p < 0.05, **p < 0.01, ***p < 0.001. Scale bar: 200 μm.

**Fig. 6:**
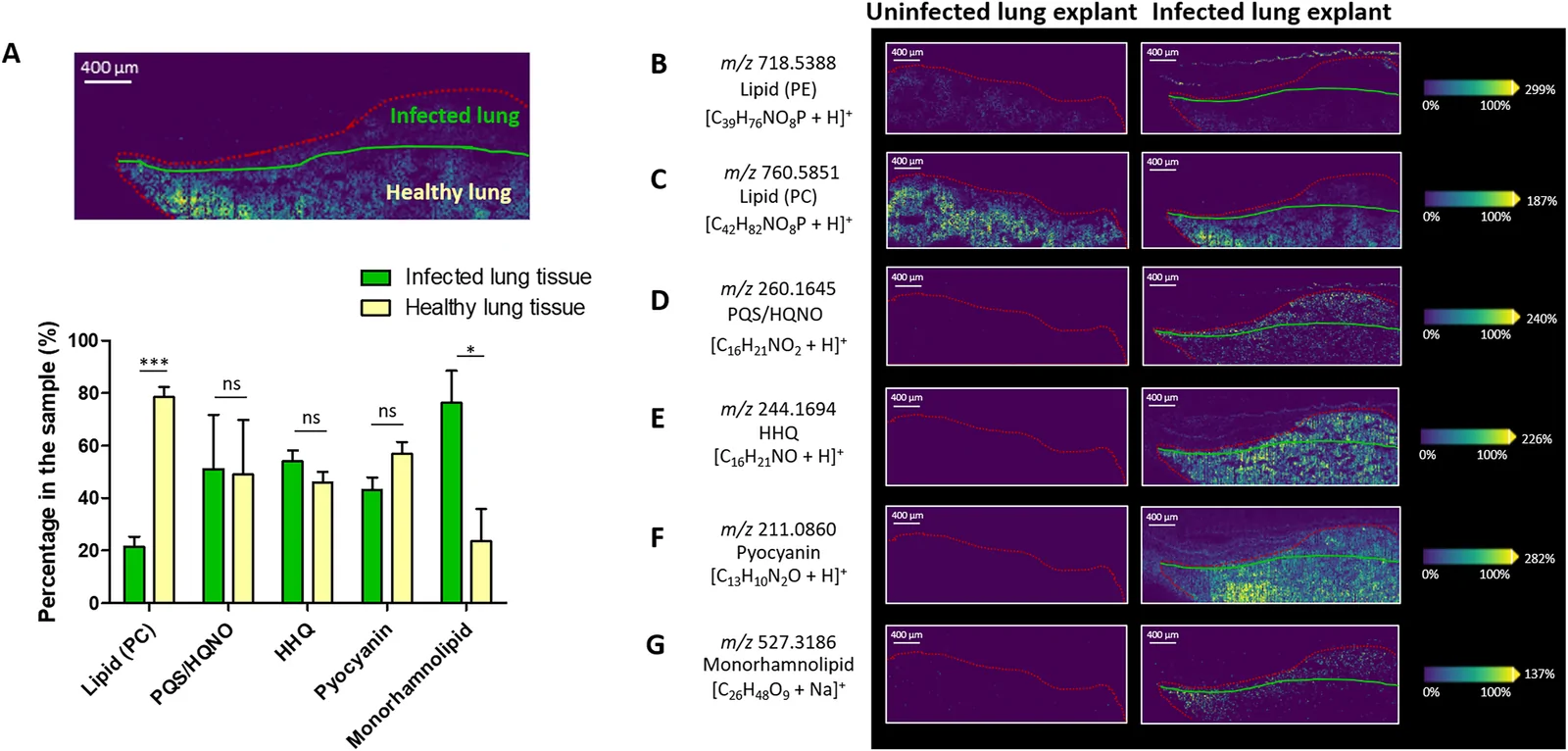
Spatial distribution of six metabolites involved in***P. aeruginosa*biofilm on infected or control lung explant.** (A) Schematic representation of infected and healthy lung tissue in *ex vivo* model and percentage of features in the sample. (B-G) Representative density ionic maps of six ions [M+H]^+^ (B-F) and [M+Na]^+^ (G) detected in infected or control lung explant after 24h. The red dotted line represents the lung explant. The green line represents the interface between infected and healthy tissue. Data are presented as mean ± SEM; Statistical analysis (n = 3) were performed using student's t-test; ns: not significant; *p < 0.05, **p < 0.01, ***p < 0.001. Scale bar: 400 μm.

Following optimization of the experimental conditions and validation of biofilm formation, untargeted MALDI-MSI analyses were performed to characterize the spatial distribution of metabolites within biofilms in both the *in vitro* model (Fig. 5) and the *ex vivo* model (Fig. 6). With the MSI configuration used in this study, data were acquired with very high precision, with the MS measurement error remaining below 3 ppm. This analytical performance ensures high reliability in determining the exact masses and, consequently, robust annotation of detected compounds. Furthermore, tandem mass spectrometry (MS/MS) analyses were also performed as soon as signal intensity allowed the obtention of an informative MSMS spectrum, in order to obtain *in situ* fragmentation profiles (Figures S2, S3 and S4).

Based on these data, we started to look for ions that could reliably discriminate between bacterial and lung tissue regions. Two lipids features were then selected for their clear and contrasting spatial distributions in both compartments to facilitate delineation of bacterial biofilm and lung tissue in the MSI sections. A first ion with a particularly interesting distribution was detected at *m/z* 718.5388 ([C_39_H_76_NO_8_P + H]^+^) and assigned to a phosphatidylethanolamine (PE) based on its MS/MS fragmentation (annotation level 3) (Figure S2 A and B). This PE showed a preferential spatial distribution in *P. aeruginosa in vitro* biofilms (Fig. 5A and B) and also enabled localization of bacterial biomass within the tissue section (Fig. 6B). This observation is consistent with the fact that PE are well known to be the predominant phospholipids in bacterial membranes [32]. On the other hand, another lipid ion detected at *m/z* 760.5851 ([C_42_H_82_NO_8_P + H]^+^), putatively assigned to a phosphatidylcholine (PC) based on its MS/MS fragmentation (annotation level 3) (Figure S2 C and D), was predominantly detected in eukaryotic cells (Fig. 6C) compared to bacterial cells (Fig. 5C) and allowed clear delineation of lung tissue. It is consistent with the fact that PC is the major phospholipid class in pulmonary surfactant [33] although PC could also be produced by *P. aeruginosa* [34]. This lipid was present in both control and infected lung explants but showed reduced intensity at the infected area where the biofilm developed (Fig. 6A and C). The reduced intensity of *m/z* 760.5851 in infected regions may reflect changes in the pulmonary lipid compartment during infection, particularly in relation to *P. aeruginosa*'s ability to hydrolyze host lipids using phospholipases. Metabolites derived from phosphocholine metabolism, such as fatty acids and choline, can also be utilized by *P. aeruginosa* as carbon sources and contribute to metabolic adaptation. Furthermore, previous studies have shown that PC availability can influence regulatory pathways involved in the metabolism and virulence of *P. aeruginosa* [35,36]. Thus, the decrease in PC signal observed in MSI could be associated with increased utilization or metabolism remodeling of *m/z* 760.5851 lipid during infection. However, imaging data do not allow to distinguish between the direct utilization of PC derived metabolites, host lipid remodeling or other biological processes contributing to the local decrease in PC signal intensity.

Despite the structural and physiological differences between the *in vitro* PDMS biofilm model and the *ex vivo* lung explant system, the metabolomic profiles showed substantial overlap, with an important part of detected metabolites shared between both conditions. The identified compounds were predominantly assigned to three major functional classes: QS molecules, phenazines, and rhamnolipids.

Within the QS network, the *m/z* 260.1645 feature was detected in both models and was putatively assigned to PQS or HQNO, two isomers with the same molecular formula (annotation level 4). They could not be distinguished by MS/MS fragmentation because of low signal intensity. This feature exhibited a significative preferential localization in a deeper biofilm layer of the *in vitro* model, observed across all three biological replicates consistent with regions of elevated bacterial density (Fig. 5A and D). Although the representative MSI image showed an enrichment of PQS/HQNO at an interfacial location in the *ex vivo* model, quantitative analysis across replicates indicated that PQS/HQNO could also be detected in deeper tissue zones (Fig. 6A and D). PQS is involved in QS and regulates the transcription of *P. aeruginosa* virulence genes [37] whereas HQNO is an inhibitor of the respiratory chain of bacteria and can promote biofilm formation by cell autolysis [38]. The precise role of *m/z* 260.1645 feature could not be determined in our model conditions. Their biosynthetic common precursor, HHQ, was detected at *m/z* 244.1694 and annotated based on its accurate mass and MS/MS spectral match against standard spectra (annotation level 2) (Fig. S3). HHQ displayed a broader spatial distribution and was detected throughout the sample in both models and across all biological replicates (Fig. 5A and E and Fig. 6A and E), consistent with its role as an extracellular signaling molecule [39]. The presence of these molecules suggests that the alkyl quinolone pathway is active in the biofilm. The absence of homoserine lactone signals such as C4-HSL and 3-oxo-C12-HSL is consistent with previous reports showing that these molecules are primarily produced during early stages of biofilm formation, whereas PQS signaling becomes dominant in mature biofilms [40].

Additional members of alkyl quinolone pathway and N-oxidized derivatives (AQNO), including NHQ, UHQ and NQNO were also detected and putatively annotated (annotation level 4) (data not shown). Especially NQNO showed spatial co-localization with PQS/HQNO, suggesting coordinated production within the quinolone signaling pathway [41].

Phenazine metabolites were detected in both experimental systems, notably including pyocyanin and 5,10-dihydrophenazine. Pyocyanin was detected at *m/z* 211.0860 and annotated based on its accurate mass and MS/MS spectral match against standard spectra (annotation level 2) (Fig. S4). In the PDMS *in vitro* model, the quantitative analysis suggests a preferential localization in the deeper biofilm layers and at the interface with PDMS across all biological replicates (Fig. 5A and F). Although not statistically significant, this trend towards preferential accumulation in basal regions suggests a strong association with zones of high cell density and potentially reduced oxygen availability, conditions known to influence phenazine production and redox activity [42]. These redox-active molecules can serve as alternative electron acceptors in oxygen-limited regions, thereby maintaining metabolic activity [43]. In contrast, within the *ex vivo* lung explant model and in all biological replicates, pyocyanin displayed a distinct distribution pattern characterized by extensive penetration into the pulmonary tissue (Fig. 6A and F). Rather than being confined to surface-associated bacterial aggregates, the molecule diffused deeply into the tissue matrix, indicating a high capacity for tissue infiltration. Pyocyanin is known to induce oxidative stress and impair immune defenses, which may contribute to the extensive tissue damage observed in infected explants [44]. Notably, the most intense pyocyanin signal, in the representative image, is detected in a region distant from biofilm, suggesting that it may act as a precursor in tissue undergoing degradation and may contribute to the early stage of tissue alteration. However, it is important to note that pyocyanin may not be the only factor involved in cellular damage during *P. aeruginosa* infection. Furthermore, the data presented in this study do not allow for a link to be established between the distribution of pyocyanin and the observed cellular damage.

Given the biological relevance of PQS/HQNO, HHQ and pyocyanin, their ionization behavior and spatial specificity under the experimental conditions was assessed by matrix-matched standard spotting experiments. Each standard was spotted at 50 μM onto control lung sections of *ex vivo* model prior to matrix deposition, on three consecutive slices by swapping their position to minimize biological variability. The resulting MALDI-MSI analysis showed that PQS, HHQ and pyocyanin standards produced the expected *m/z* feature and characteristic MS/MS fragments under experimental conditions (Fig. S5). The signal of expected *m/z* features was specifically increased at the spotting locations (Fig. S5). These results validate that those three metabolites can be efficiently ionized under *ex vivo* model conditions and support the specificity of their spatial distribution observed in biological samples. Contrarily, HQNO was poorly detected in the lung tissue due to its lower ionization efficiency, at the concentration studied, under *ex vivo* model conditions.

Rhamnolipids are well-known biosurfactants produced by *P. aeruginosa*. The *m/z* 527.3186 feature was putatively assigned to mono-rhamnolipid species (C10-C10 congeners), based on its accurate mass (annotation level 4). Due to low intensity, its structural identity could not be determined by MS/MS fragmentation. It was consistently identified in both experimental models, highlighting its conserved production across *in vitro* and *ex vivo* conditions. Spatial mapping suggested a preferential localization of this biosurfactant at the interface between bacterial communities and their surrounding environment although with substantial inter-replicate variability (Fig. 5A and G and Fig. 6A and G). This localization agrees with its well-documented physicochemical properties. These amphipathic molecules play an important role in biofilm architecture and dynamics [45]. By reducing surface tension, rhamnolipids facilitate bacterial motility and contribute to the spatial organization of the biofilm matrix [46]. Its presence therefore indicates an actively structured and dynamic biofilm.

Overall, the combined detection of QS signals, phenazines, and rhamnolipids highlights the existence of a highly coordinated molecular network within *P. aeruginosa* biofilms. QS regulates collective bacterial behavior, phenazines sustain energy metabolism under oxygen limitation, and rhamnolipids contribute to structural organization. Together, these metabolites support biofilm persistence, environmental adaptation, and virulence during host colonization.

The successful detection and spatial localization of these molecules confirm the relevance of the two developed models for MSI-based investigation of biofilm biology and host–pathogen interactions.

## 4 Conclusion

Two innovative models have been developed to mimic different chronic biofilm infection situations. The original cross-section design allows all layers of the biofilm to be studied simultaneously, thereby visualizing the homogeneous or heterogeneous distribution of molecules within the biofilm. These models are easily modifiable and can therefore be adapted to different strains, culture media, incubation times, supports, etc. In particular, including biofilm-related mutant strains or temporal MSI analyses during biofilm development could provide further insights into the dynamics of biofilm formation and host-pathogen interactions. Coupling the models with MSI, for which these were developed and optimized, revealed a conserved molecular profile for both types of biofilms despite the different experimental conditions. Overall, the spatial distribution of metabolites observed in this study highlights the highly organized metabolic architecture of *P. aeruginosa* biofilms. QS molecules act as global regulators that coordinate bacterial communication and control the expression of virulence factors. Phenazines, including pyocyanin, establish a redox-active environment that supports bacterial metabolism under oxygen-limited conditions while also contributing to oxidative stress in surrounding tissues. Rhamnolipids participate in the structuring and dynamics of the biofilm matrix, facilitating surface colonization and biofilm expansion. Rather than acting independently, these metabolites form an interconnected regulatory and metabolic network that enables the biofilm to persist, adapt to environmental constraints and interact with host tissues. The pronounced presence and diffusion of pyocyanin in the lung explant model further illustrates how molecules primarily involved in biofilm physiology may also contribute directly to host damage during infection. By preserving spatial information, MALDI-MSI therefore provides a powerful approach to simultaneously investigate biofilm organization, metabolic activity and host-pathogen interactions.

## CRediT authorship contribution statement

**Marie Droniou:** Writing – review & editing, Writing – original draft, Software, Methodology, Investigation, Formal analysis. **Clément Guillou:** Writing – review & editing, Writing – original draft, Supervision, Software, Methodology, Investigation, Formal analysis. **Hung Le:** Writing – review & editing, Writing – original draft, Supervision, Software, Methodology, Investigation, Funding acquisition, Formal analysis, Conceptualization. **Christophe Arnoult:** Writing – review & editing, Writing – original draft, Methodology. **Teddy Grandjean:** Writing – review & editing, Writing – original draft. **Philippe Gosset:** Writing – review & editing, Writing – original draft. **Pascal Cosette:** Writing – review & editing, Writing – original draft, Supervision, Methodology, Funding acquisition, Conceptualization. **Isabelle Schmitz:** Writing – review & editing, Writing – original draft, Supervision, Software, Methodology, Funding acquisition, Data curation, Conceptualization.

## Declaration of competing interest

The authors declare the following financial interests/personal relationships which may be considered as potential competing interests: Pascal Cosette reports financial support was provided by Defeating Cystic Fibrosis Association. If there are other authors, they declare that they have no known competing financial interests or personal relationships that could have appeared to influence the work reported in this paper.

## Data Availability

The mass spectrometry imaging data have been deposited to the ProteomeXchange Consortium via the PRIDE partner repository with the dataset identifier PXD079719.
