# Supplementary material for: Exploring the spatial distribution of biomolecules in Pseudomonas aeruginosa biofilms formed on PDMS and lung explants by mass spectrometry imaging
Source: Biofilm. 2026 Sep 17;12:100401. doi: 10.1016/j.bioflm.2026.100401 (PMC13602144; doi:10.1016/j.bioflm.2026.100401)
Supplement: Supplementary file 1 [file mmc1.docx]

**Figure S1: Optimization of biofilm embedding.** Mirror plot comparing Agar, CMC, OCT and gelatin mass spectra under MALDI conditions with DHB matrix and DHB spectra alone at *m/z* 100-500 and *m/z* 500-1500 windows.

*m/z*

*m/z*

x10^3^

x10^4^

*Intensity*

*Intensity*

***m/z 5*00-1500**

***m/z* 100-500**

x

x

x

*1

*1

**MS/MS**

*m/z* 760.6 ± 0.25

NCE = 15

*1

*1

PE Head group

Chemical Formula : C_2_H_8_O_4_NP

Exact Mass : 141.0191

*1

*1

**MS/MS**

*m/z* 718.6 ± 0.25

NCE = 15

*1

**[C_39_H_76_NO_8_P+H]^+^**

*m/z* 718.5381 (∆ppm = 2.6)

*1

**PhosphoCholine (PC) Head group**

**DAG fragment**

(*m/z* 577.5181)

**(Loss of PE Head group)**

Chemical Formula : C_5_H_15_O_4_NP^+^

Exact Mass : 184.0733

**B**

**D**

**Colonized PDMS**

**Infected lung explant**

**C**

**A**

**[C_42_H_82_NO_8_P+H]^+^**

*m/z* 760.5851 (∆ppm = 1.2)

**Figure S2: Annotation of two lipids with tandem mass spectrometry .** (A) MS Spectra in positive-ion mode with a mass resolution of 140,000 at *m/z* 200, at 700-840 *m/z* mass range of colonized PDMS with a focus on [C_39_H_76_NO_8_P+H]^+^ *m/z* 718.5362 (Red square). (B) Experimental MS/MS spectra for hypothetical identified lipid: [C_39_H_76_NO_8_P+H]^+^. (C) MS Spectra in positive-ion mode with a mass resolution of 140,000 at *m/z* 200, at 700-840 *m/z* mass range of colonized PDMS with a focus on [C_39_H_76_NO_8_P+H]^+^ *m/z* 760.5860 (Red square). (D) Experimental MS/MS spectra for hypothetical identified lipid: [C_42_H_82_NO_8_P+H]^+^. *Blue crosses indicate fragment ions attributed to analyzer-related signals, as these ions were also observed when fragmenting background noise*. **NCE: Normalized Collision Energy**.

159.0675

172.0754

**A**

**HHQ**

***m/z* 244.1689**

**MS/MS Spectra**

*1

*2

**Infected lung explant**

**B**

Absent in uninfected lung explant

∆ppm = 2.8

*m/z* 244.1689

**MS/MS**

**C**

*m/z* 244.2 ± 0.25

NCE = 60

244.1692

*1

*2

**Figure S3: Annotation of HHQ with tandem mass spectrometry .** (A) Molecular representation of HHQ (*m/z* 244.1689) and its predicted MS/MS spectra in positive-ion mode (from PubChem). (B) MS Spectra in positive-ion mode with a mass resolution of 140,000 at *m/z* 200, at 180-275 *m/z* mass range of infected lung explant with a focus at 244.00-244.25 *m/z* (right grey square). Left grey square represents the same focus in MS spectra of uninfected lung explant as negative control. (C) Experimental MS-MS spectra for HHQ with specific molecular signature (represented by *1 and *2). NCE: Normalized Collision Energy.

211.0859

**B**

*3

*2

*1

Absent in uninfected lung explant

196.0624

183.0912

168.0677

**MS/MS Spectra**

**Infected lung explant**

**Pyocyanin**

***m/z* 211.0866**

∆ppm = 2.8

*m/z* 211.0860

**A**

**C**

*2

*3

***2**

*1

***3**

***1**

211.0857

**MS/MS**

*m/z* 211.1 ± 0.25

NCE = 50

**Figure S4: Annotation of pyocyanin with tandem mass spectrometry .** (A) Molecular representation of pyocyanin (*m/z* 211.0866) and its standard MS/MS spectra in positive-ion mode (*in situ* onto *ex vivo* lung section). (B) MS Spectra in positive-ion mode with a mass resolution of 140,000 at *m/z* 200, at 180-275 *m/z* mass range of infected lung explant with a focus at 211.90-211.20 m/z (left grey square). Right grey square represents the same focus in MS spectra of uninifected lung explant as negative control. (C) Experimental MS-MS spectra for pyocyanin with specific molecular signature (represented by *1, *2 and *3). NCE: Normalized Collision Energy.

**Figure S5: Matrix-matched standard spotting validation.** Spatial distribution and MS/MS fragmentation of HHQ, pyocyanin, PQS and HQNO (50 µM ; 0.2 µL) spotted onto *ex vivo* lung model section. White-dotted line represents the lung section. No detection of HQNO at the given amount chosen for the study. *HHQ signal is observed instead of HQNO as it is present as interference in the standard.* **NCE: Normalized Collision Energy. Scale : 400 µm**.

*m/z* 244.1695

(HHQ)

2905

0

*m/z* 211.0863

(Pyocyanin)

2905

0

*m/z* 260.1645

(PQS/HQNO)

2905

0

*

*

*

*

*

*

*

*

**HHQ**

**Pyocyanin**

**PQS**

**HQNO**

400 µm

**NCE = 60**

**NCE = 60**

**NCE = 50**
